# A New Cellulose-Based Fluorescent Probe for Specific and Sensitive Detection of Cu^2+^ and Its Applications in the Analysis of Environmental Water

**DOI:** 10.3390/polym14112146

**Published:** 2022-05-25

**Authors:** Fei Zhao, Zhiyuan Meng, Zhonglong Wang, Yiqin Yang

**Affiliations:** Jiangsu Co-Innovation Center of Efficient Processing and Utilization of Forest Resources, College of Light Industry and Food, Nanjing Forestry University, Nanjing 210037, China; xx@njfu.edu.cn (F.Z.); mengzhiyuan@nifu.edu.cn (Z.M.)

**Keywords:** carboxymethyl cellulose, fluorescent probe, microspheres, Cu^2+^

## Abstract

In this work, a novel fluorescent probe CMC−GE−AQ with an effective sensitive detection ability for Cu^2+^ was synthesized and constructed by using carboxymethyl cellulose (CMC) as the skeleton and 8-aminoquinoline (AQ) as the fluorophore. This probe exhibited a highly specific “turn-off” fluorescence response to Cu^2+^, and the fluorescence color changed from bright orange to colorless after adding Cu^2+^. The probe could selectively detect Cu^2+^ in a complex environment and its detection limit (LOD), the binding constant (K_a_) and the numbers of binding sites (n) were calculated to be 6.4 × 10^−8^ mol L^−1^, 1.7 × 10^6^ mol^−1^ L and 1.2, respectively. The sensing detection mechanism was confirmed by X-ray photoelectron spectroscopy (XPS) and density functional theory (DFT) calculations. In addition, the probe CMC−GE−AQ was successfully applied to detect Cu^2+^ in real water samples, and CMC−GE−AQ-based fluorescent microspheres can serve as a convenient tool for the detection of Cu^2+^.

## 1. Introduction

Cu^2+^, as the third essential trace element in the human body, plays an important role in biological processes by participating in the activities of cells [1]. However, high level of Cu^2+^ in the body can cause great harm to human health, such as depression, diarrhea, memory loss and other symptoms, and can even lead to Hashimoto’s disease, fibrocystic breast disease, Alzheimer’s disease, Wilson’s disease, etc. [2,3]. Furthermore, a high concentration of Cu^2+^ is a common water pollutant, with toxicity, non-degradation and bioaccumulation [4,5], which can enter the food chain and into the human body through water circulation. Therefore, the detection of Cu^2+^ is necessary for environmental protection and human health.

The detection methods of Cu^2+^ include atomic absorption spectrometry, atomic emission spectrometry, inductively coupled plasma spectrometry, visible spectrophotometry, chromatography, etc. [6,7,8,9,10,11]. These conventional methods have some problems such as expensive testing equipment, difficult operations and long detection time. Compared with the above methods, fluorescence spectroscopy can well overcome the above defects and is widely used in the detection of Cu^2+^ [12,13,14,15,16,17,18,19]. Hence, it is necessary and significant to develop fluorescent probes for monitoring Cu^2+^.

Carboxymethyl cellulose (CMC), as a kind of water-soluble natural polymer, is usually found in the form of sodium salts with the advantages of excellent biocompatibility, nontoxicity, good biodegradability and easy modification [20]. Due to the presence of many active oxygen-containing groups (carboxyl and hydroxyl), CMC is also an ideal carrier in the field of multifunctional modifications and constructing diverse fluorescent materials [21,22,23,24], For example, Fan prepared fluorescent hydrogels and aerogel hybrid materials by covalent coordination of lanthanide ions (Eu^3+^ or Tb^3+^) with carboxyl groups of CMC, which can be used to the detection of Fe^3+^ [23]. Ye synthesized a fluorescent probe CMC/Tb(III) for detecting Mn^2+^ in aqueous solution [24]. Thus, it can be seen that CMC-based fluorescent probes have great application prospects due to their high sensitivity and selectivity, good processability, and operability.

8-aminoquinoline (AQ), as a kind of fluorescent compound with multiple N donor atoms, can chelate with metal ions. Recently, AQ-based probes have attracted much attention in the field of detecting metal ions [25,26,27,28,29,30]. Fu synthesized a fluorescent probe for Zn^2+^ detection by condensation reaction of 8-aminoquinoline derivative and 4-(diethylamino)salicylaldehyde [29]. Wang designed a colorimetric probe based on an aminoquinoline derivative to detect Cu^2+^ in water [30].

In this work, a novel carboxymethyl cellulose-based fluorescent probe CMC−GE−AQ toward Cu^2+^ detection was prepared. This probe was designed by immobilizing AQ onto CMC with epichlorohydrin. The CMC−GE−AQ solution exhibited bright orange fluorescence, and the fluorescence was immediately quenched after the addition of Cu^2+^. CMC−GE−AQ could detect Cu^2+^ with high sensitivity. The detection limit (LOD), the binding constant (K_a_) and the numbers of binding sites (n) were calculated to be 6.4 × 10^−8^ mol L^−1^, 1.7 × 10^6^ mol^−1^ L and 1.2, respectively. In addition, CMC−GE−AQ could monitor Cu^2+^ in real water samples, and CMC−GE−AQ-based fluorescent microspheres were successfully prepared for the detection of Cu^2+^.

## 2. Materials and Methods

### 2.1. Materials

Sodium carboxymethyl cellulose (CMC−Na, viscosity: 300~800 mPa⋅s) was purchased from Sinopharm Chemical Reagent Co., Ltd. (Shanghai, China). Epichlorohydrin (ECH), sodium hydroxide (NaOH), 8-aminoquinoline (AQ), tetrahydrofuran (THF), span 80, liquid paraffin, butyl alcohol, carbon tetrachloride, N,N-dimethylformamide (DMF) and ethylene diamine tetraacetic acid (EDTA) were acquired from Nanjing Chemical Reagent Co., Ltd. (Naning, China). The stock solutions of metal ions were prepared from various metal salts (CuSO_4_, Pb(NO_3_)_2_, AgNO_3_, HgSO_4_, ZnCl_2_, CdSO_4_, MgSO_4_, AlCl_3_, CsCl and MnSO_4_). All of these reagents were used without further purification. All solutions were prepared with deionized water.

### 2.2. Experimental Methods

#### 2.2.1. Preparation of Carboxymethyl Cellulose Glycidyl Ether (CMC−GE)

First, 2 g CMC−Na was completely dissolved in 40 mL deionized water, and the pH was adjusted to 14 with 30 wt% NaOH solution. Then, 8 mL of epichlorohydrin (ECH) was added dropwise into the CMC−Na solution to react at 60 °C for 3 h. After the reaction, the mixture was separated out by ethanol and filtered, and the residues were washed with ethanol and distilled water until neutrality to obtain CMC−GE. The epoxy group of CMC−GE was determined according to the literature [31].

#### 2.2.2. Preparation of Probe (CMC−GE−AQ)

First, 2 g CMC−GE was added to 75 mL of water, and the pH of CMC−GE solution was adjusted to 12. Then, 18 g AQ was dissolved in a certain amount of THF, and the AQ solution was added into the CMC−GE solution, accompanied by stirring. The mixture reacted at 65 °C for 6 h. The reacted mixture was separated out by ethanol and filtered, the residue was washed with ethanol and distilled water to remove the unreacted AQ completely (the filtrate was colorless and no fluorescence). The remainder was 3-(quinolin-8-amino)-2-hydroxypropyl carboxymethyl cellulose ether (CMC−GE−AQ).

#### 2.2.3. Characterization

The structural analyses of samples were recorded by the FT-IR spectra with a scan range from 4000 to 650 cm^−1^ using an infrared spectrometer (VERTEX 80 V, Bruker). Thirty-two scans were recorded with a resolution of 4 cm^−1^.

The XPS spectra were performed in an X-ray photoelectron spectrometer (AXIS UltraDLD, Shimadzu, Kyoto, Japan) using a monochromatized Al Ka X-ray source (1486.6 eV). The spectral acquisition range was from 1100 to 0 eV, with an energy of 160 eV and a scan step of 1.0 eV. The high-resolution XPS spectra and quantitative analysis data of CMC−GE−AQ of C 1s, O 1s and N 1s were recorded. The quantitative relative sensitivity factors (RSF) of C 1s, O 1s and N 1s were 0.278, 0.780 and 0.477, respectively.

The UV–Vis absorption spectra of CMC−GE−AQ were determined by a Shimadzu UV-2450 spectrophotometer, and the scan range was from 225 to 350 nm.

The fluorescence spectra of testing samples were recorded by fluorescence spectrophotometer (LS 55, PE Co., Norwalk, CT, USA); the excitation wavelength was at 425 nm and excitation slit was at 7 nm.

The color changes of testing samples were observed under a 365 nm UV lamp. The Cu^2+^ concentrations were measured by atomic absorption spectrometry (TAS−990AFG, Beijing, China).

Gaussian 09 program calculated using the B3LYP function with the 6−31G* set.

The surface morphologies of CMC−GE−AQ-based fluorescent microspheres were determined with field emission scanning electron microscopy (FESEM) using a Regulus 8100 (Hitachi, Tokyo, Japan) at 100 magnifications.

The CMC−GE−AQ-based fluorescent microspheres were imaged by a confocal laser scanning microscope (LSM710, Zeiss, Jena, Germany).

#### 2.2.4. Fluorescence Properties Testing of CMC−GE−AQ

Preparation of the test solvent: Probe CMC−GE−AQ was dissolved in 2 mL DMF/H_2_O (*v*/*v* = 8/2) to obtain 2 mL CMC−GE−AQ solution (concentrations 1.5 × 10^−4^ g mL^−1^, pH = 7). To evaluate the fluorescence performance of CMC−GE−AQ, the fluorescence spectra of CMC−GE−AQ solution in the presence of various 10^−4^ mol L^−1^ metal ions (Pb^2+^, Ag^+^, Hg^2+^, Zn^2+^, Cd^2+^, Mg^2+^, Al^3+^, Cs^+^, Mn^2+^ and Cu^2+^) and different concentrations of Cu^2+^ (0~10^−4^ mol L^−1^) were recorded. To study the reversibility of CMC−GE−AQ for Cu^2+^ detection, Cu^2+^ and the strong chelating agent EDTA were alternately added to CMC−GE−AQ solution; the fluorescence intensity of CMC−GE−AQ with Cu^2+^ (10^−4^ mol L^−1^) and EDTA (1.5 × 10^−4^ mol L^−1^) was recorded.

The detection limit of CMC−GE−AQ for Cu^2+^ was calculated by using emission titration datum. The emission spectra of the original CMC−GE−AQ were measured 20 times. Then, the relationship between the fluorescence emission intensity of the CMC−GE−AQ at 579 nm and the concentration of Cu^2+^ was plotted. Each fluorescence emission intensity was measured 3 times. The detection limit (LOD) was calculated as Equation (1):

(1) $$\mathrm{LOD}=\frac{\;3\sigma \;}{s}$$

where σ is the standard deviation of the emission intensity of the original CMC−GE−AQ, and s is the slope between the fluorescence emission intensity and the concentration of Cu^2+^.

#### 2.2.5. Real Water Samples Testing

The real water samples used in this experiment were Yangtze river water, Xuanwu lake water and tap water, which had been filtered and removed from impurities. Then, the samples were tested by the following steps: Probe CMC−GE−AQ was dissolved in DMF/real water samples (*v*/*v* = 8/2) to obtain CMC−GE−AQ solution (concentrations = 1.5 × 10^−4^ g mL^−1^, pH = 7), and then the same amount of 2 μL of 10^−3^ mol L^−1^ Cu^2+^ was gradually added into 2 mL CMC−GE−AQ solution (Cu^2+^ concentrations = 0, 1, 3, 5 and 7 × 10^−6^ mol L^−1^), and the Cu^2+^ concentrations were determined by fluorescence spectroscopy. In addition, the same amount of 2 μL of 10^−3^ mol L^−1^ Cu^2+^ was gradually added into 2 mL DMF/real water samples (*v*/*v* = 8/2) solution (Cu^2+^ concentrations = 0, 1, 3, 5 and 7 × 10^−6^ mol L^−1^), and the Cu^2+^ concentrations were determined by atomic absorption spectrometry for comparison.

#### 2.2.6. Preparation of CMC−GE−AQ-based Microspheres

The CMC−GE−AQ-based microspheres were prepared according to the literature [32]. When 0.63 g CMC−GE−AQ was completely dissolved in 12.5 mL of 30 wt% NaOH solution, the CMC−GE−AQ solution was added into the mixture of liquid paraffin (50 mL), Span 80 (0.2 g), t-butanol (0.7 mL) and CCl_4_ (0.7 mL) dropwise, accompanied by high-speed blending. Then, 2.5 mL of epichlorohydrin (ECH) was added dropwise into the mixture to react at 60 °C for 6 h. After the reaction, the mixture was separated out by ethanol and filtered, and the residues were washed with ethanol and distilled water until neutrality to obtain CMC−GE−AQ-based microspheres.

## 3. Results and Discussion

### 3.1. Synthesis and Characterization of CMC−GE−AQ

**Scheme 1:**
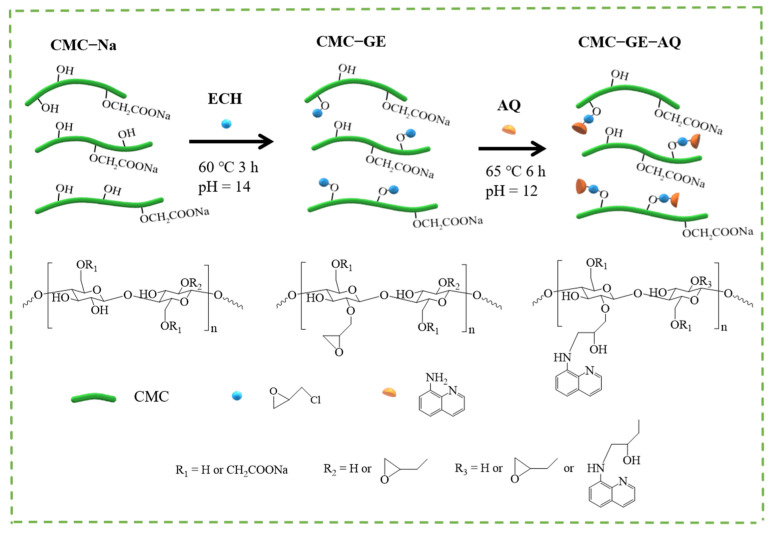
Synthetic route of the probe CMC−GE−AQ.

The synthetic route of CMC−GE−AQ is shown in Scheme 1. The reaction between the hydroxyl group of CMC−Na and ECH usually required alkaline conditions [33]. The fluorophore group was introduced onto the CMC−GE chain by the ring-opening reaction of epoxy groups with amino groups of AQ [34].

**Figure 1:**
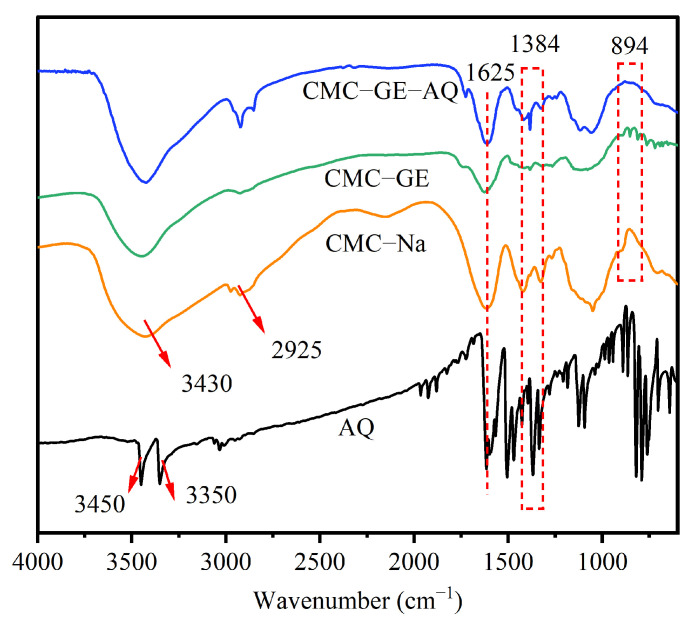
FT-IR spectra of CMC−GE−AQ, CMC−GE, CMC−Na and AQ.

The FT-IR spectra of CMC−GE−AQ, CMC−GE, CMC−Na and AQ are compared in Figure 1. The FT-IR spectra of AQ showed that absorptions at 3450 and 3350 cm^−1^ were primary amine stretching [35]. Absorptions at 3430 and 2925 cm^−1^ were found in CMC−GE−AQ, CMC−GE and CMC−Na. The former was O−H stretching vibration, and the latter was −CH_2_− stretching vibration. The absorptions of CMC in the range of 1000~1300 cm^−1^ were C−O−C stretching vibrations [33]. The FT-IR spectra of CMC−GE showed an obvious characteristic absorption of epoxy groups at 894 cm^−1^ [32]. After AQ was functionalized, the characteristic absorption of the epoxy group almost disappeared, and the absorptions at 1384 and 1625 cm^−1^ originated from C−N stretching vibration and N−H stretching vibration, respectively [36]. The FT-IR spectra data strongly elucidated that the AQ was introduced onto CMC−GE by the epoxy groups.

**Figure 2:**
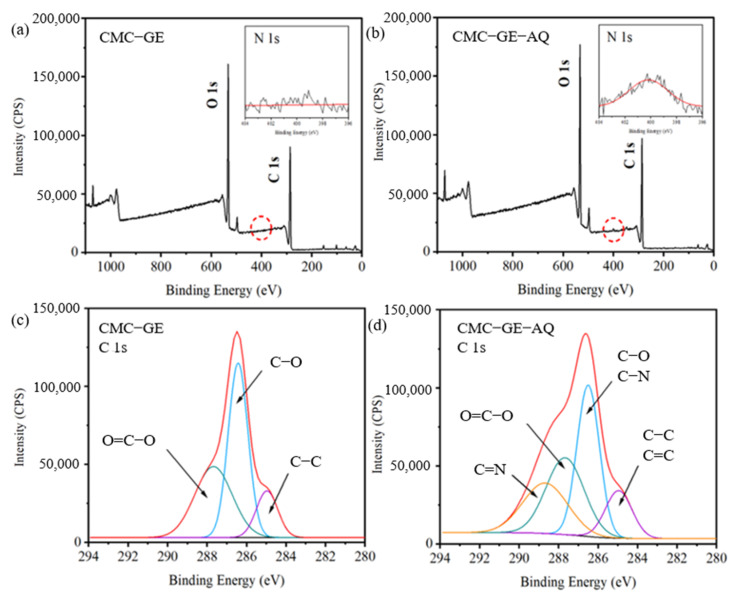
XPS spectra of (**a**,**c**) CMC−GE and (**b**,**d**) CMC−GE−AQ. (Inset: High-resolution XPS spectra of the N 1s peak. (**c**,**d**) High-resolution XPS spectra of the C 1s peak.)

The covalent load of AQ on the CMC−GE surface was further confirmed by XPS. The XPS spectra of CMC−GE and CMC−GE−AQ are shown in Figure 2. In Figure 2a, only C 1s and O 1s absorption were found in CMC−GE. In Figure 2b, C 1s, O 1s, and N 1s absorption were found in CMC−GE−AQ. In Figure 2c, the absorptions of CMC−GE are located at 284.96, 286.56 and 287.76, which are C−C, C−O, and O−C=O bonds, respectively. In Figure 2d, the peak of CMC−GE−AQ found at 288.76 eV appeared because of the C=N bond. The XPS analysis survey scans of CMC−GE and CMC−GE−AQ are shown in Table 1. The N content (1.44%) appeared in CMC−GE−AQ after the introduction of AQ, and C content changed from 56.51% to 57.39%. Thus, the conclusion of XPS confirmed that AQ was successfully introduced to CMC−GE.

**Figure 3:**
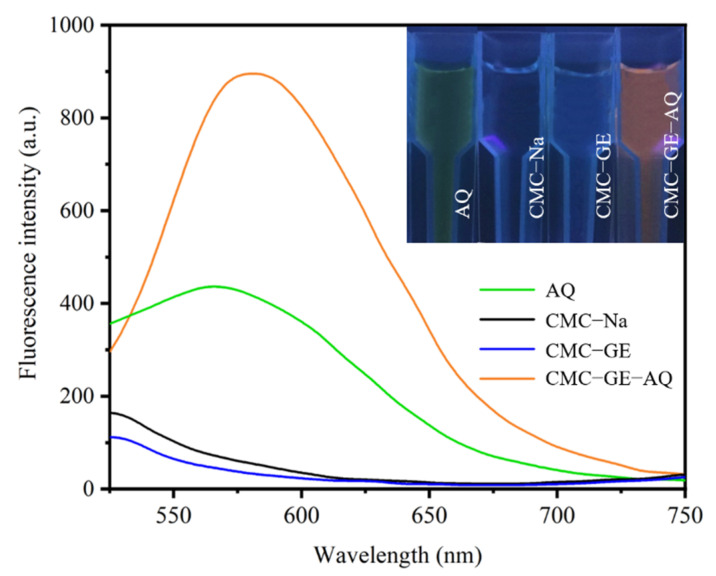
Fluorescence spectra and (inset) fluorescence photographs of AQ, CMC−Na, CMC−GE and CMC−GE−AQ solution. (CMC−GE−AQ: 1.5 × 10^−4^ g mL^−1^; solvents: DMF/H_2_O solution (*v*/*v* = 8/2, pH = 7); excitation wavelength: 425 nm; excitation slit: 5 nm.)

The fluorescence spectra and fluorescence photographs of AQ, CMC−Na, CMC−GE and CMC−GE−AQ are shown in Figure 3, and the concentrations of all the sample solutions were 1.5 × 10^−4^ g mL^−1^. The CMC and CMC−GE solution had no fluorescence. AQ solution showed weak green fluorescence at 565 nm, while CMC−GE−AQ solution showed strong orange fluorescence at 579 nm. When AQ was attached to CMC−GE, the fluorescence intensity of CMC−GE−AQ was about two times that of AQ, and the emission wavelength redshifted and the color changed significantly. According to the structural characteristics of CMC−GE−AQ, the enhanced fluorescence intensity could be related to the CMC skeleton. CMC−GE−AQ improved the weakness of the weak fluorescence emission intensity of AQ. Therefore, AQ was successfully introduced to CMC−GE.

### 3.2. Effect of Detection Conditions on Fluorescence Intensity of CMC−GE−AQ

The fluorescence intensity of CMC−GE−AQ was greatly affected by the environmental medium. This study focused on the effect of solvent polarity and pH value on fluorescence intensity of CMC−GE−AQ.

#### 3.2.1. Solvents Polarity

**Figure 4:**
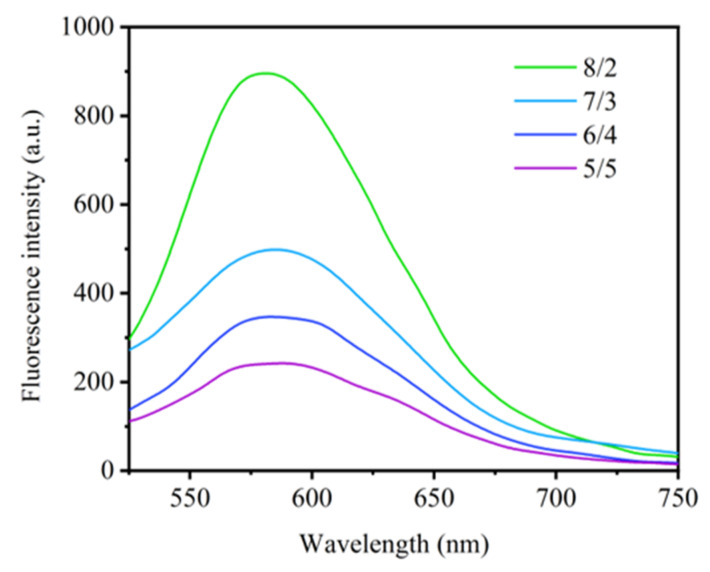
Fluorescence spectra of CMC−GE−AQ in DMF/H_2_O solvents with different DMF proportions. (CMC−GE−AQ: 1.5 × 10^−4^ g mL^−1^; solvents: pH = 7; excitation wavelength: 425 nm; excitation slit: 5 nm.)

The highly polar solvent ensured the fluorescence intensity of fluorescent materials, such as water, DMF, DMSO, DMAc, etc. [37]. Therefore, the mixture of DMF and water was selected as the detection solvent to study the effect of solvent on the fluorescence intensity of CMC−GE−AQ (Figure 4). With the increase in DMF amount, the fluorescence intensity of CMC−GE−AQ at the emission peak gradually increased, which was because water has a high dielectric constant (εr = 80.1) and more hydrogen bonds, while DMF has a low dielectric constant (εr = 36.7) and no hydrogen bonds [31]. With the increase in DMF ratio, the polarity of the solvent decreased and the dielectric constant decreased. Therefore, the fluorescence intensity of CMC−GE−AQ increased with the decrease in solvent polarity, and the increase trend was consistent with the decrease in the average dielectric constant [38]. The maximum fluorescence emission was obtained at a volume ratio DMF/H_2_O of 8/2. Therefore, the mixed solvent of DMF/H_2_O (*v*/*v* = 8/2) was selected as the detection solvent for subsequent experiments.

#### 3.2.2. Solvents pH Value

**Figure 5:**
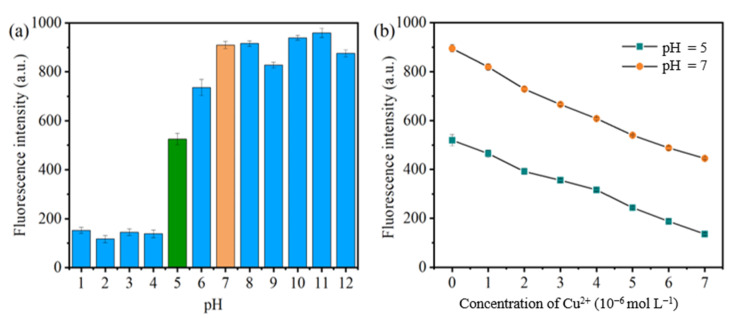
(**a**) Fluorescence intensity of CMC−GE−AQ solution at 579 nm at different pH values. (**b**) Fluorescence intensity of CMC−GE−AQ solution at the pH value 5 and 7 with different Cu^2+^ concentrations. (CMC−GE−AQ: 1.5 × 10^−4^ g mL^−1^; solvents: DMF/H_2_O solution (*v*/*v* = 8/2); Cu^2+^: 0~7 × 10^−6^ mol L^−1^; excitation wavelength: 425 nm; excitation slit: 5 nm.)

As shown in Figure 5a, when the solvent of pH < 5, the CMC−GE−AQ solution showed weak fluorescence intensity. When the solvent of pH ranged from 5 to 7, the fluorescence intensity of CMC−GE−AQ solution increased significantly. When the solvent of pH > 7, the fluorescence intensity of CMC−GE−AQ solution remained at a high level. The suitable pH detection range of CMC−GE−AQ was 5~12. Different concentrations of Cu^2+^ were added to the CMC−GE−AQ solution at the pH values 5 and 7, respectively. The relationship between fluorescence intensity and Cu^2+^ concentration is shown in Figure 5b. The fluorescence intensity of CMC−GE−AQ solution (pH = 7) decreased with the increase in the concentration of Cu^2+^, which was consistent with the trend of CMC−GE−AQ solution (pH = 5). Since Cu^2+^ did not hydrolyze at pH = 5, it also did not hydrolyze at pH = 7. Therefore, the pH value 7 of the CMC−GE−AQ solvent system was selected for subsequent metal ions detection.

Based on the above factors, the suitable detection conditions of CMC−GE−AQ for metal ions was as follows: Volume ratio of DMF/H_2_O was 8/2, and solvent pH was 7.

### 3.3. Fluorescence Responses to Various Metal Ions

**Figure 6:**
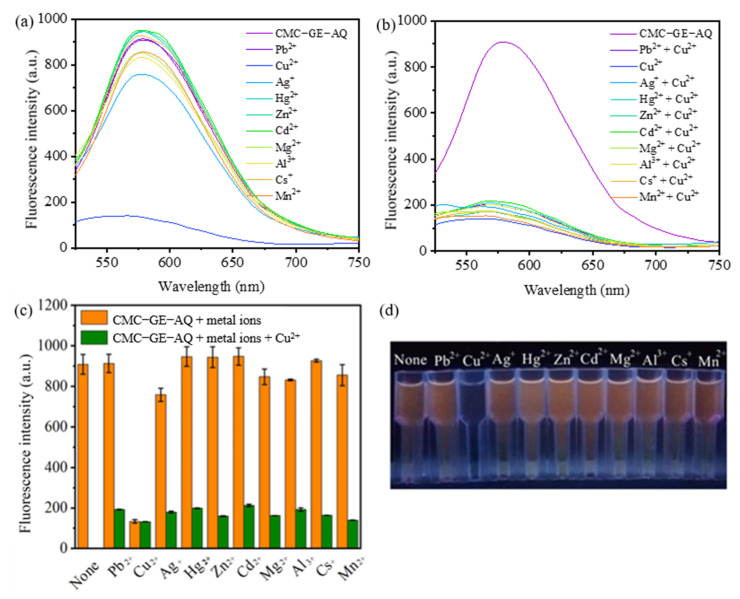
(**a**,**b**) Fluorescence spectra and (**c**) maximum fluorescence intensity of CMC−GE−AQ solution with different metal ions and other metal ions coexisting with Cu^2+^. (**d**) Fluorescence photographs of CMC−GE−AQ solution with different metal ions under 365 nm UV lamp. (CMC−GE−AQ: 1.5 × 10^−4^ g mL^−1^; solvents: DMF/H_2_O solution (*v*/*v* = 8/2, pH = 7); metal ions: 10^−4^ mol L^−1^; excitation wavelength: 425 nm; excitation slit: 5 nm.)

The fluorescence selectivity of CMC−GE−AQ solution (1.5 × 10^−4^ g mL^−1^) with different metal ions (10^−4^ mol L^−1^) in shown in Figure 6. Among the 10 metal ions, most of them had no significant effects on the fluorescent phenomenon of CMC−GE−AQ, while only Cu^2+^ significantly influenced the fluorescence emission, causing fluorescence quenching (Figure 6a). Thus, the results showed that the CMC−GE−AQ could be used as a novel florescence sensor for identifying Cu^2+^. When CMC−GE−AQ reacted with Cu^2+^, it was not affected by inner−filter effects. In order to study the anti−interference ability of CMC−GE−AQ to Cu^2+^ detection, the fluorescence emission spectra of CMC−GE−AQ were recorded when other metal ions coexisted with Cu^2+^ (Figure 6b). As seen in Figure 6c, when other metal ions coexist with Cu^2+^, the fluorescence intensity of CMC−GE−AQ solution changed slightly. In Figure 6d, among the 10 metal ions, fluorescence quenching was performed after the interaction between Cu^2+^ and CMC−GE−AQ solution. Hence, those results indicated that CMC−GE−AQ could be a fluorescence−quenching probe for Cu^2+^ with good selectivity and anti-interference.

### 3.4. Fluorescence and UV–Vis Responses of CMC−GE−AQ for Cu^2+^

**Figure 7:**
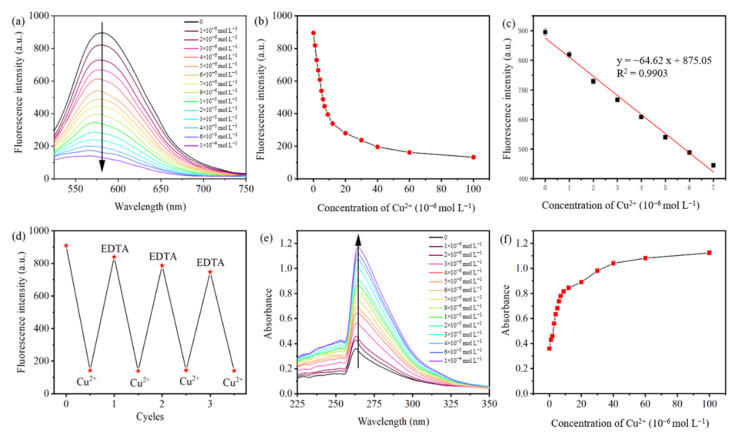
(**a**) Fluorescence spectra and (**b**) maximum emission intensity changed at 579 nm of CMC−GE−AQ solution with different concentrations of Cu^2+^ (0~10^−4^ mol L^−1^). (**c**) Relationship between fluorescence intensity at 579 nm and different Cu^2+^ concentrations (0~7 × 10^−6^ mol L^−1^). (**d**) The reversible changes of fluorescence intensity of CMC−GE−AQ to Cu^2+^ for 3 cycles. (**e**) UV-Vis absorption spectra and (**f**) maximum absorbance changed at 263 nm of CMC−GE−AQ solution with different concentrations of Cu^2+^ (0~10^−4^ mol L^−1^). (CMC−GE−AQ: 1.5 × 10^−4^ g mL^−1^; Solvents: DMF/H_2_O solution (*v*/*v* = 8/2, pH = 7); Excitation wavelength: 425 nm; Excitation slit: 5 nm).

The concentration dependence of CMC−GE−AQ on Cu^2+^ under fluorescence and UV conditions was studied. Figure 7a presents the fluorescence intensity of CMC−GE−AQ solution (1.5 × 10^−4^ g mL^−1^) with different Cu^2+^ concentrations (0~10^−4^ mol L^−1^), and the fluorescence intensity of CMC−GE−AQ was reduced with the increase in Cu^2+^ concentration. In Figure 7b, with the increase in Cu^2+^ concentrations (0~7 × 10^−6^ mol L^−1^), the maximum fluorescence intensity of CMC−GE−AQ fell quickly, and when the concentration of Cu^2+^ was higher than 7 × 10^−6^ mol L^−1^, this decline became slow. In Figure 7c, the maximum fluorescence intensity of CMC−GE−AQ showed a good linear relationship (R^2^ = 0.9903) with concentrations of Cu^2+^ in the range of 0~7 × 10^−6^ mol L^−1^. According to Equation (1), the LOD of CMC−GE−AQ for Cu^2+^ was as low as 6.4 × 10^−8^ mol L^−1^, which is lower than other probes that have been reported for the detection of Cu^2+^ (Table 2). Therefore, CMC−GE−AQ could be used as a sensitive quenching probe to detect Cu^2+^ at the micromolar level. Figure 7d shows the fluorescence intensity of CMC−GE−AQ solution (1.5 × 10^−4^ g mL^−1^) after 3 cycles of Cu^2+^ (10^−4^ mol L^−1^) and EDTA (1.5 × 10^−4^ mol L^−1^). The fluorescence intensity of CMC−GE−AQ recovered to 82% of the initial intensity after three cycles, indicating that CMC−GE−AQ is a reversible probe for Cu^2+^. In general, the fluorescence quenching process could be divided into static quenching and dynamic quenching [39]. In order to distinguish them, the UV–Vis absorption spectra of CMC−GE−AQ (1.5 × 10^−4^ g mL^−1^) with different amounts of Cu^2+^ (0~10^−4^ mol L^−1^) were measured and illustrated in Figure 7e. With the increase in Cu^2+^ concentrations, the UV–Vis absorption peak of CMC−GE−AQ at 263 nm was enhanced (Figure 7f), indicating that the quenching effect of Cu^2+^ on CMC−GE−AQ was attributed to a static quenching mechanism, and these absorption peak changes should be caused by the complexation of CMC−GE−AQ with Cu^2+^ [39].

### 3.5. Detection Mechanism Study

**Figure 8:**
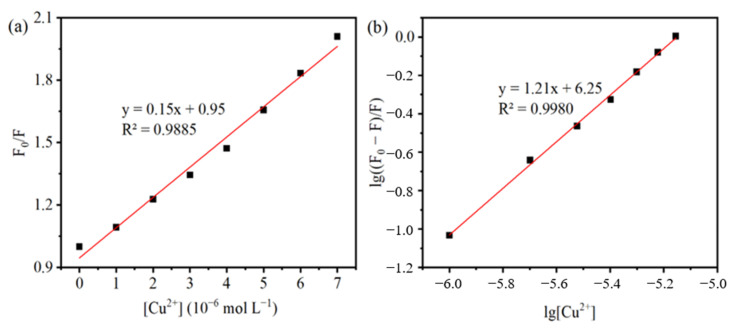
(**a**) The Stern–Volmer and (**b**) lg((F_0_ − F)/F)~lg[Cu^2+^] plots of CMC−GE−AQ with Cu^2+^. (CMC−GE−AQ: 1.5 × 10^−4^ g mL^−1^; solvents: DMF/H_2_O solution (*v*/*v* = 8/2, pH = 7); excitation wavelength: 425 nm; excitation slit: 5 nm.)

The static quenching was studied in terms of the Stern–Volmer equation shown in Equation (2) [39]. In addition, the binding constants and number of binding points could be used to determine whether the interaction between CMC−GE−AQ and Cu^2+^ formed a complex. It is assumed that Cu^2+^ has an independent number of binding sites (n) on CMC−GE−AQ. The apparent binding constant (K_a_) and the number of binding sites (n) was determined based on Equation (3) [48]:

(2) $$\frac{F_{0}}{F}{=1+K}_{s}[{\mathrm{Cu}}^{2+}]$$

(3) $$\mathrm{lg}\frac{{(F}_{0}-\;F)}{F}{=\mathrm{lgK}}_{a\;}{+\mathrm{nlg}[\mathrm{Cu}}^{2+}]$$

where F_0_ and F are the fluorescence intensity of CMC−GE−AQ in the absence and presence of Cu^2+^, respectively. [Cu^2+^] is one the of concentrations of Cu^2+^. K_s_ and K_a_ are the static quenching constant of the Stern–Volmer equation and the binding constant, respectively. n is the number of binding sites of CMC−GE−AQ and Cu^2+^. The linear relation of F_0_/F versus [Cu^2+^] is shown in Figure 8a. Based on Equation (2), K_s_ was calculated to be 1.5 × 10^5^ mol^−1^ L. The linear relation of lg((F_0_ − F)/F) versus lg[Cu^2+^] is displayed in Figure 8b. Based on Equation (3), K_a_ and n were obtained to be 1.7 × 10^6^ mol^−1^ L and 1.2, respectively. The high correlation coefficient (R^2^ = 0.998) indicated that the assumption proposed was reasonable. Moreover, K_a_ was greater than 10^4^ mol^−1^ L, indicating that CMC−GE−AQ had a strong binding ability with Cu^2+^, and n was close to 1, suggesting that there was one binding site for CMC−GE−AQ toward Cu^2+^.

**Figure 9:**
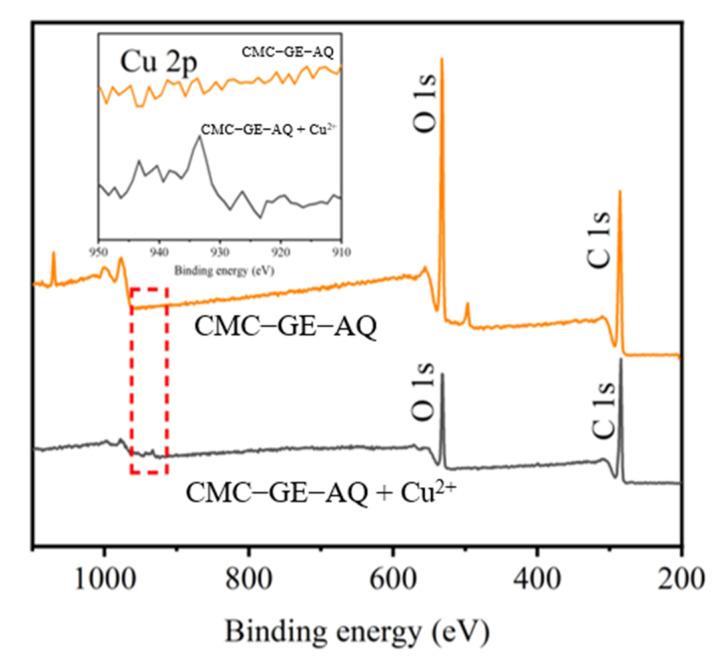
The XPS spectra of the CMC−GE−AQ and CMC−GE−AQ + Cu^2+^ complexes.

The recognition mechanism of CMC−GE−AQ for Cu^2+^ was investigated by XPS analysis and DFT calculations. As shown in Figure 9, after the reaction of CMC−GE−AQ with Cu^2+^, the absorption of O 1s decreased significantly, and the absorption of CMC−GE−AQ at 497 eV vanished after the reaction with Cu^2+^. In addition, the CMC−GE−AQ + Cu^2+^ complex showed a weak absorption at 933.34 eV, which originated from the characteristic absorption of Cu^2+^ [49]. Therefore, the O atom on CMC−GE−AQ was involved in the complexation of Cu^2+^.

**Figure 10:**
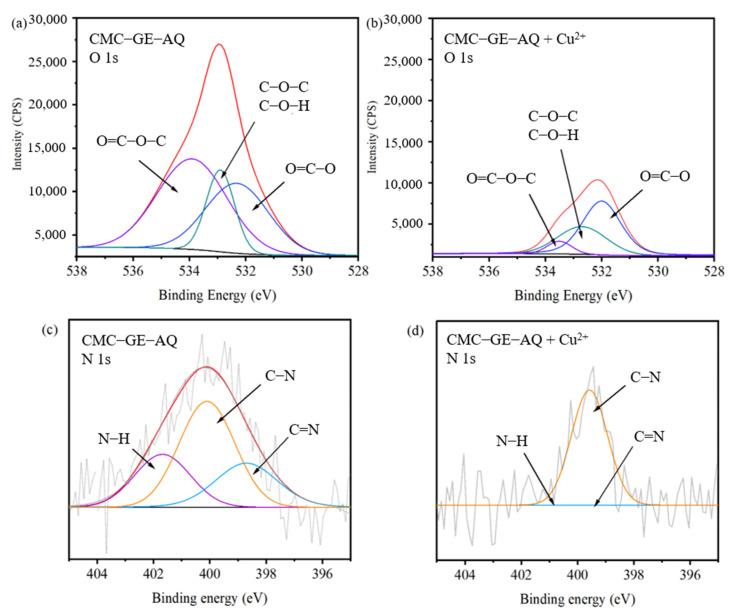
High resolution XPS spectra of (**a**,**b**) O 1s and (**c**,**d**) N 1s of CMC−GE−AQ and CMC−GE−AQ + Cu^2+^.

According to the High-resolution XPS spectra of O 1s (Figure 10a,b). The three absorptions at 532.3, 533.0 and 533.9 eV originated from the absorptions of O=C−O, C−O−C/C−O−H and O=C−O−C, respectively. Compared with CMC−GE−AQ and the CMC−GE−AQ + Cu^2+^ complex, C−O−C/C−O−H contents at 533.0 eV decreased and O=C−O−C contents at 533.9 eV markedly decreased. It can be concluded that the oxygenic groups structure of CMC−GE−AQ was involved in the complexation with Cu^2+^. The High-resolution XPS spectra of N 1s (Figure 10c,d) showed that the absorptions of CMC−GE−AQ could be divided into three main absorptions at 398.7, 400.2 and 401.7 eV respectively, corresponding to C=N, C−N and N−H bonds, respectively. After the reaction with Cu^2+^, the peak strength of the C=N and N−H bonds weakened, indicating that the N atom of the CMC−GE−AQ was involved in the complexation of Cu^2+^.

**Figure 11:**
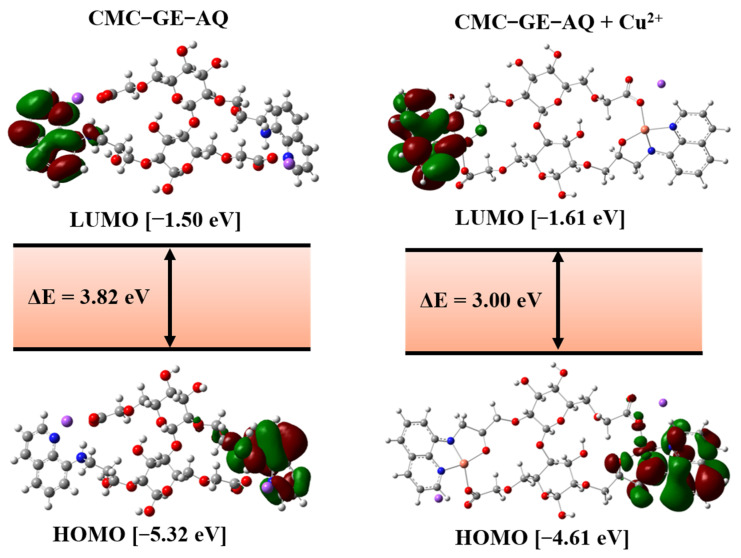
HOMO, LUMO and HOMO-LUMO energy gaps of the CMC−GE−AQ and CMC−GE−AQ + Cu^2+^ complexes.

Density functional theory (DFT) calculations of the CMC−GE−AQ and CMC−GE−AQ + Cu^2+^ complexes were performed by the Gaussian 09 program. The optimal structures of the CMC−GE−AQ and CMC−GE−AQ + Cu^2+^ complexes along with their highest occupied molecular orbital (HOMO) and lowest unoccupied molecular orbital (LUMO) are depicted in Figure 11. The HOMOs of CMC−GE−AQ were mainly distributed on the naphthalene ring and hydroxyl, and the LUMOs were distributed on the whole naphthalene ring. The HOMOs of the CMC−GE−AQ + Cu^2+^ complex were mainly distributed on the naphthalene ring and oxygen−containing functional groups (carboxyl and hydroxyl), while the LUMOs were distributed on the whole naphthalene ring. Therefore, after CMC−GE−AQ interacted with Cu^2+^, the electrons were transferred from the carboxyl to the naphthalene ring, which would lead to the change in fluorescence, and the sensing mechanism of the interaction between CMC−GE−AQ and Cu^2+^ was a photoinduced electron transfer (PET) [14,16]. After the interaction between CMC−GE−AQ and Cu^2+^, the HOMO-LUMO energy gaps changed from 3.82 to 3.00 eV, becoming more stable. This may be due to the fact that the coordination with metal made the structure of CMC−GE−AQ more planar, thus increasing the stiffness of the CMC−GE−AQ [50].

**Figure 12:**
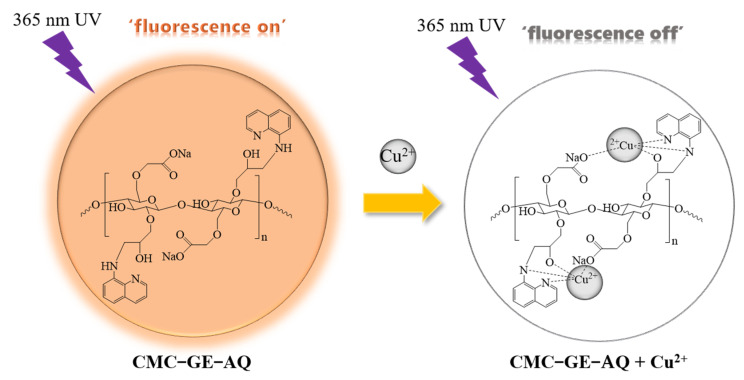
Sensing mechanism of CMC−GE−AQ with Cu^2+^.

To sum up, the Cu^2+^ sensing process of CMC−GE−AQ is shown in Figure 12. The recognition sites of CMC−GE−AQ were located at N−H, C=N, O=C−O−C and −OH, and the sensing mechanism was a combination of photoinduced electron transfer (PET) and metal ion chelation [51].

### 3.6. Application of CMC−GE−AQ

This study focused on the environmental and materialized application of CMC−GE−AQ. The probe CMC−GE−AQ could be used to detect Cu^2+^ in real water samples. Based on the good processing performance of carboxymethyl cellulose, CMC−GE−AQ prepared into portable microspheres also had the ability to detect Cu^2+^.

#### 3.6.1. Application in Real Water Samples

**Figure 13:**
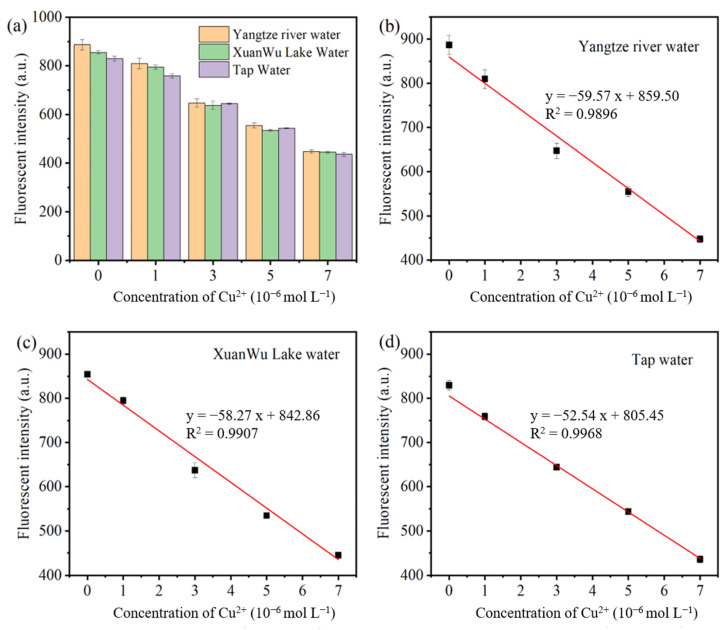
(**a**) Fluorescence intensity of CMC−GE−AQ at 579 nm for different concentrations of Cu^2+^; a linear relationship between fluorescence intensity at 579 nm and Cu^2+^ concentration in (**b**) Yangtze river water, (**c**) Xuanwu lake water and (**d**) tap water. (CMC−GE−AQ: 1.5 × 10^−4^ g mL^−1^; solvents: DMF/real water samples solution (*v*/*v* = 8/2, pH = 7); Cu^2+^: 0, 1, 3, 5 and 7 × 10^−6^ mol L^−1^; excitation wavelength: 425 nm; excitation slit: 5 nm).

With the development of modern industry, the discharge of a large number of industrial wastewater containing Cu^2+^ has caused serious water pollution, which not only has seeped into the groundwater, but has also flowed into rivers and lakes. Therefore, three real water samples (Xuanwu Lake water, Yangtze River water and tap water) were selected to detect Cu^2+^. As shown in Figure 13, the fluorescence intensity of CMC−GE−AQ solution at 579 nm showed a good linear relationship with the concentration of Cu^2+^ (0, 1, 3, 5 and 7 × 10^−6^ mol L^−1^). The concentrations of Cu^2+^ were calculated according to the fitting equation of CMC−GE−AQ solution and were measured by atomic absorption spectrometry, as shown in Table 3. Compared with the recovery of Cu^2+^ measured by atomic absorption spectrometry (112.0%~183.0%), the recovery of Cu^2+^ measured by CMC−GE−AQ (82.0%~118.7%) was closer to 100%. These results indicate that the probe CMC could detect Cu^2+^ in environmental real water samples.

#### 3.6.2. Application of CMC−GE−AQ-based Microspheres

**Figure 14:**
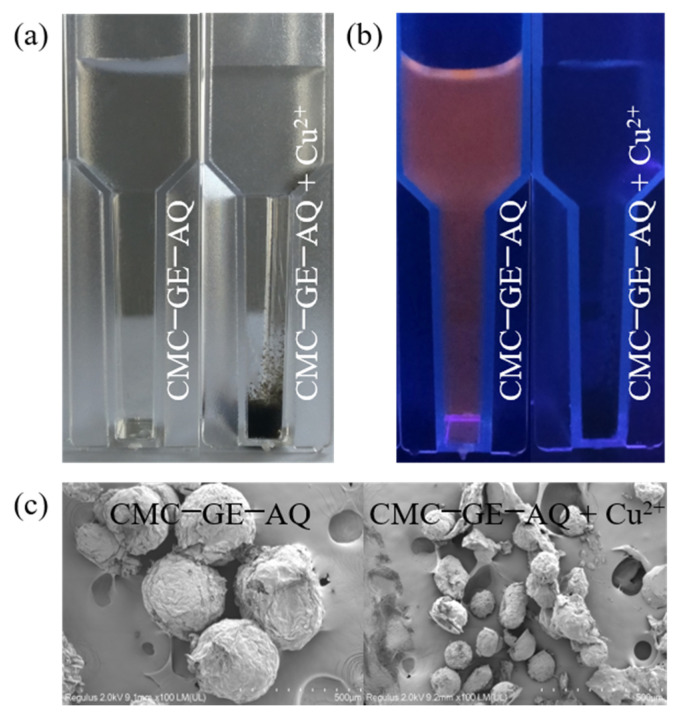
(**a**) Digital (visible light) and (**b**) fluorescent (365 nm) photographs and (**c**) SEM images of CMC−GE−AQ microspheres with 1.5 × 10^−3^ mol L^−1^ Cu^2+^.

CMC−GE−AQ-based microspheres were prepared to expand the application of this probe. CMC−GE−AQ microspheres could be uniformly dispersed into the DMF/H_2_O (*v*/*v* = 8/2) solvent system (1.5 × 10^−4^ g mL^−1^, pH = 7) with 1.5 × 10^−3^ mol L^−1^ Cu^2+^. In Figure 14a, CMC−GE−AQ microspheres all aggregated and sank to the bottom, turning black in color and decreasing in size. In Figure 14b, under a 365 nm UV lamp, CMC−GE−AQ microspheres suspension showed bright orange fluorescence and fluorescence quenching after interacting with Cu^2+^. In Figure 14c, CMC−GE−AQ microspheres had obvious aggregation phenomena after the interaction with Cu^2+^, and the size of the microspheres decreased from 400 to 100 μm. Therefore, CMC−GE−AQ microspheres could be used as a fluorescence sensor of Cu^2+^ and could recognize Cu^2+^ in aqueous solution with the naked eye.

**Figure 15:**
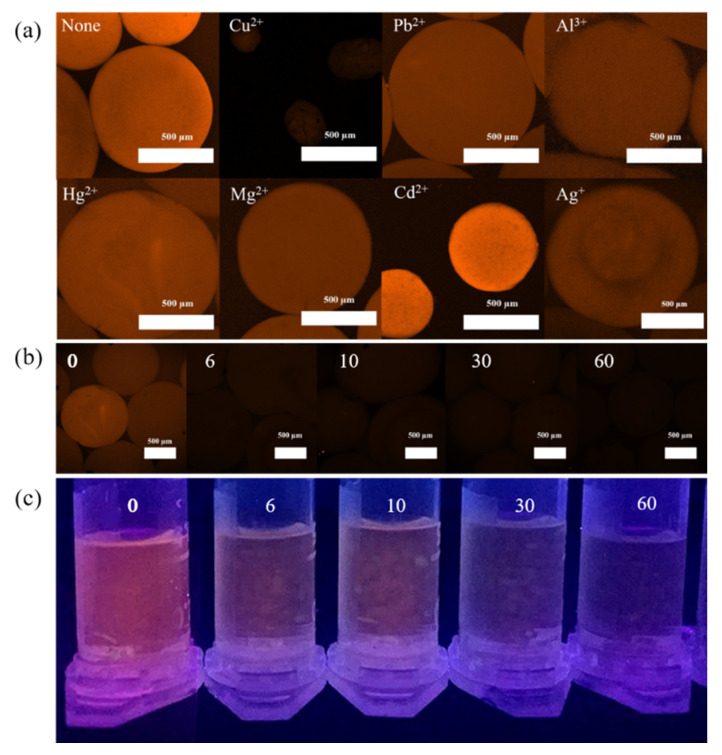
CLSM of CMC−GE−AQ microspheres with (**a**) several typical metal ions (10^−4^ mol L^−1^) and (**b**) different concentrations of Cu^2+^ (0, 6, 10, 30 and 60 × 10^−6^ mol L^−1^); (**c**) fluorescent photographs of CMC−GE−AQ suspension with different concentrations (0, 6, 10, 30 and 60 × 10^−6^ mol L^−1^) under 365 nm UV lamp. (CMC−GE−AQ: 1.5 × 10^−4^ g mL^−1^; solvents: DMF/H_2_O solution (*v*/*v* = 8/2, pH = 7); excitation wavelength: 425 nm; excitation slit: 5 nm).

CMC−GE−AQ microspheres were added into DMF/H_2_O (*v*/*v* = 8/2) solution (1.5 × 10^−4^ g mL^−1^, pH = 7) with several typical metal ions and different concentrations (0~60 × 10^−6^ mol L^−1^) of Cu^2+^. Afterward, the microspheres were employed for imaging by confocal laser scanning microscope (CLSM). As shown in Figure 15a, after interacting with several typical metal ions, only Cu^2+^ could quench the fluorescence of CMC−GE−AQ suspension, indicating that the CMC−GE−AQ-based microspheres also have high selectivity for detecting Cu^2+^. As shown in Figure 15b,c the fluorescence of CMC−GE−AQ microspheres decreased continuously after adding different concentrations of Cu^2+^, which was consistent with the conclusion of fluorescence spectra. Therefore, CMC−GE−AQ-based microspheres could be used as an effective and convenient tool for Cu^2+^ sensing.

## 4. Conclusions

In summary, a simple fluorescent probe CMC−GE−AQ for the detection of Cu^2+^ was successfully prepared. Upon coordination with Cu^2+^, the fluorescent color of CMC−GE−AQ changed significantly from orange to colorless. CMC−GEj−AQ exhibited a good sensitivity, selectivity and reversibility for Cu^2+^. Its LOD for Cu^2+^ was computed to be 6.4 × 10^−8^ mol L^−1^, and K_a_ and n were obtained to be 1.7 × 10^6^ mol^−1^ L and 1.2, respectively. The detection mechanism was confirmed by XPS and DFT calculations. In addition, probe CMC−GE−AQ could monitor Cu^2+^ in real water samples. Furthermore, CMC−GE−AQ-based fluorescent microspheres could serve as an effective tool for detecting Cu^2+^. This work promoted the development of CMC in the field of fluorescent sensing.

## Figures and Tables

**Table 1:** XPS analysis survey scans of CMC−GE and CMC−GE−AQ.

Samples	C 1s %	O 1s %	N 1s %
CMC−GE	56.51	43.49	0
CMC−GE−AQ	57.39	41.17	1.44

**Table 2:** Comparison of CMC−GE−AQ with other Cu^2+^ probes.

Cu^2+^ Probes	Detection Limits (mol L^−1^)	References
Coumarin-based probe	6.4 × 10^−7^	[16]
Naphthalene conjugate BODIPY dye-based probe	1.28 × 10^−6^	[17]
Luminescent AIZS−GO nanocomposites	1.8 × 10^−7^	[40]
Nitrogen-doped carbon quantum dots (N−CQDs)	9 × 10^−8^	[41]
Chromoxanthin-based probe (TR−V)	8.4 × 10^−8^	[42]
Dibenzimidazole-based probe	9.4 × 10^−8^	[43]
BODIPY-based Turn-off probe (HHPBA−BODIPY)	3.5 × 10^−7^	[44]
Benzotriazole-based probe	8 × 10^−6^	[45]
Benzimidazole-based chemodosimeter (APBHN)	1.48 × 10^−7^	[46]
Diarylethene-derived colorimetric probe	5.47 × 10^−7^	[47]
CMC−GE−AQ	6.4 × 10^−8^	This work

**Table 3:** Measurements of Cu^2+^ in three real water samples by fluorescence spectroscopy and atomic absorption spectrometry.

Real Water Samples	Standard Added Cu^2+^ Concentrations (×10^−6^ mol L^−1^)	Standard Detected by Fluorescence Spectroscopy (×10^−6^ mol L^−1^)	Recovery by Fluorescence Spectroscopy (%)	Standard Detected by Atomic Absorption Spectrometry (×10^−6^ mol L^−1^)	Recovery by Atomic Absorption Spectrometry (%)
Xuanwu Lake water	0	Not detected	−	Not detected	−
	1	0.84 ± 0.16	84.0	1.48 ± 0.11	148.0
	3	3.56 ± 0.41	118.7	4.59 ± 0.26	153.0
	5	5.11 ± 0.10	102.2	7.36 ± 0.07	147.2
	7	6.91 ± 0.08	98.7	10.38 ± 0.04	148.3
Yangtze River water	0	Not detected	−	Not detected	−
	1	0.82 ± 0.13	82.0	1.83 ± 0.03	183.0
	3	3.52 ± 0.38	117.3	4.93 ± 0.12	164.0
	5	5.28 ± 0.21	105.6	7.59 ± 0.23	151.8
	7	6.83 ± 0.13	97.6	10.58 ± 0.04	151.1
Tap water	0	Not detected	−	Not detected	−
	1	0.87 ± 0.10	87.0	1.12 ± 0.04	112.0
	3	3.06 ± 0.05	102.0	4.35 ± 0.11	145.0
	5	4.98 ± 0.01	99.6	7.57 ± 0.08	151.1
	7	7.03 ± 0.06	100.4	10.26 ± 0.06	146.6

## Data Availability

The data presented in this study are available on request from the corresponding author.
